# Knowledge hardly translates to reality—A randomized controlled trial on sexual abuse prevention for girls with intellectual disabilities

**DOI:** 10.3389/fpsyt.2022.886463

**Published:** 2022-07-19

**Authors:** Olaf Reis, Frank Häßler, Anne Daubmann, Wencke Chodan

**Affiliations:** ^1^Department of Child and Adolescent Psychiatry and Neurology, University Medical Center Rostock, Rostock, Germany; ^2^Institute of Medical Biometry and Epidemiology, Medical Center Hamburg-Eppendorf, Hamburg, Germany

**Keywords:** child sexual abuse, intellectual disability, prevention, girls, group training

## Abstract

**Objective:**

People with intellectual disabilities (ID) are at higher risk of being sexually abused and developing psychiatric disorders in consequence. The effects of behavior prevention programs for children with ID have rarely been investigated. Previous studies suffer from methodical weaknesses, such as the lack of a control group, small sample size, or invalid outcome measures. This study aimed at demonstrating the efficacy of a prevention program by overcoming these flaws.

**Methods:**

A group prevention program was developed and evaluated. One hundred and six girls aged from 8 to 12 years with mild ID were enrolled in a randomized controlled study, comparing the training to a sham intervention. Effects were examined in a three-time follow-up design as individual changes in preventive knowledge (board game, verbal reports) and preventive behavior (role-play, *in situ* tests). Participants' behaviors were videotaped and rated by three blinded raters.

**Results:**

Girls from the intervention group (*n* = 64) showed significant improvements in preventive knowledge compared with the control group (*n* = 39) but showed non-significant improvements for preventive behavior. *In situ* tests with realistic seduction situations revealed no improvement. The intervention proved to be safe, but several risks need to be considered.

**Discussion:**

This is the first study that evaluates a behavioral prevention program on sexual abuse for children with ID on a high level of evidence. Group interventions empowering girls with ID to recognize abuse situations are suitable to enhance sexual preventive knowledge but are less suitable to enhance preventive behavior. Naturalistic settings are indispensable for providing evidence for preventive interventions in children with ID.

## Introduction

Prevention research has grown substantially during the last decades, targeting various social grievances, such as children playing with firearms, families corrupted by drugs, the elderly being battered, or girls with intellectual disabilities being sexually abused. Being sexually abused constitutes a major risk for developing a mental disorder and accompanying behavioral problems (1). Papalia et al. (2) found a 7 times increased odds ratio for victims of abuse to develop these problems, which increased to 9.8 in the case of female victims. Individuals who have suffered child sexual abuse (CSA) display symptoms as a result of the CSA (3) and are referred to mental health services more often than those who have not (4). In their 2016 report on mental health issues, the World Psychiatry Association acknowledged that persons with ID often face a “triple jeopardy” composed of ID, abuse, and mental illnesses, the last being a result of the first two. In many cases, increased likelihoods to develop mental problems after CSA are amplified by additional problems, such as experiencing violence as an adult (5).

People with intellectual disabilities (ID) are particularly vulnerable to sexual abuse, even more so if they live in institutions (6). Most victimized women with ID were abused before their 18th birthday (7, 8), which makes girls and female teenagers with ID a highly vulnerable target group for secondary preventive health care and prevention research. According to a recent meta-analysis (9), the probability of CSA is somewhat smaller than the numbers found by Baladerian (7) who had detected that CSA has been experienced in 40–85% of women with ID. The study presented here followed the recommendation drawn from the prevalence rates found by Baladerian (7), which urge to build preventive skills at an early age. Modern prevention studies increasingly include RCT-designs in everyday situations, thereby providing more and more evidence-based legitimation for decisions about the allocation of resources (10). Despite these advances, prevention studies have not yet gained attention (1), compared to studies about consequences of CSA. One reason for this lack of attention perhaps is that prevention research often failed to meet the strict criteria of clinical studies. In eight studies on adults and five studies on children targeting sexual abuse preventive skills trainings for individuals with ID, we found methodological weaknesses, which we have listed before [(11); list of reviewed studies in the appendix] and found no study on a sample larger than 15 participants. In short, all studies on children with ID known to us suffer at least of one of the shortcomings listed here: (a) no RCT, (b) sample too small to detect generalizable effects, (c) skills training not tailored to the needs of younger people with ID, (d) outcomes poorly measured, e.g., rated by trainers themselves, (e) small generalizability of effects, e.g., preventive behavior not measured in naturalistic settings or for a few situations only. Situations of CSA in naturalistic settings vary widely in method (e.g., coercion vs. lure), or offenders (e.g., age, sex, social relation to the victim). Many of these shortcomings are due to the smallness, the youngness and the obscureness of the field which lacks not only a theory of preventive behavior for people with ID but also lacks consistent ideas about the “ideal” outcome. Seduction situations vary widely in terms of power, institutional framing (e.g., church vs. family) or trust. However, this variation is hard to describe since there is only scarce research about the distribution or structure of sexual abuse. Already Sobsey and Doe (12) described some patterns of sexual abuse of people with ID based on a sample drawn from different service centers and varying in age, such as (1) most cases of abuse were committed by men against women, (2) sexual abuse varied in terms of coercion and physical contact, (3) victims were less likely to withdraw from situations or to report to trustees, (4) about 10% of perpetrators were female, 5) perpetrators varied greatly in age [about 10% of perpetrations are committed by other children and adolescents (13); younger perpetrators, however, were shown to prefer older victims (14)], (6) in more than a half of all incidents, victims and perpetrators had some kind of relationship, (7) offenses took place more often in private (>50%) than in public places (<10%). Thus, different kinds of behavior vary in adequacy for the victim to stay safe and healthy in the best possible way.

The aim of the present study was to evaluate a program developed to teach groups of girls with ID preventive skills empowering them to recognize and to cope with seduction situations and situations of sexual abuse. Both the training and the evaluation were designed to avoid as many shortcomings from the list above as possible. To do that, we followed guidelines distilled from the prevention literature, listed in the following:

- In the dark field of CSA, the strong wish to see positive changes should not bias the researchers, meaning that the team would try their best to empower the participating girls, but should be open to all ends, negative results included. In a broader context, there are no bad results in prevention research (10), because prevention as a social duty cannot be canceled but should be effectively re-directed.
- With the absence of a theory or a model of preventive behavior, we would try to measure both, preventive knowledge and preventive behavior, not knowing how these constructs may be related. Methods of data acquisition should be tailored to the sample and stay reliable [see (15)].
- To obtain generalizable data on preventive behavior, *in situ* probes within real settings have been established, temporarily deceiving the participants for the greater good [see (15)]. Despite the resistance to be expected from officials or parents, the actors playing the offender or the trainers, we decided to go this way in a quest to achieve the highest validity possible. Videotaping the original reactions of girls allowed us to present the material to blind raters.
- For children with ID, behavioral interventions should be preferred, because interventions solely based on knowledge might exceed their capacities—and because protective behaviors, such as refusals of unwanted touches, need to be reinforced and valued (16). Behavioral skills trainings have been successfully applied several times to children with ID [see (15)]. Therefore, the training applied here contains elements of pure knowledge acquisition as well as increasingly behavioral elements.
- Situational effects were assumed to play an important role. For example, an abuse embedded into the trusted institution of the church will be deciphered differently than grooming within public spaces. The inclusion of this factor into the model, however, would have exceeded the resources of the project. That is why we decided to control situational effects during the training by block-random assignment of situations to persons.
- The training should contain different modules addressing sexual knowledge, social and self- awareness, self-assertiveness, and the chain of preventive behaviors “do not engage—say no—tell the offender to leave or leave yourself—report to a trusted person” [see (17)]. The aim of the study presented here was to prove the effectiveness of the training as a whole. The evaluation of the effectiveness of different modules would have required a much larger sample.
- To ensure the comprehensiveness of the training and the manual, it should be discussed constantly with non-academic experts from the field. For that reason, a professional association (e.g., a non-governmental organization) should be included into the study.
- The study aims at empowering girls with ID. Several studies have shown that girls become victimized much more often than boys (7, 18). The experts in the focus groups stated that prevention trainings for boys should include the topic of actively crossing boundaries and contain potential offender prevention [compare (18, 19)]. With limited resources available, this study focused on the most vulnerable group.
- Close contact between female participants and the trainer was assumed to be effective for any kind of prevention, therefore the training was to be tested against a sham intervention. The sham intervention was similar to the training in length and intensity of contact between trainer and group, but different in content.

Of the training programs we found in the literature, only a few (20); addressing children or young teenagers with ID have been evaluated. The training program developed for this study processed experiences from these studies and tried to meet the requirements as they were put together from the literature by Martinello (16). For example, children with ID have problems to differentiate between abuse and care, are mostly inapt to report, need personal care and assistance, are not sexually educated but are often surrounded by fear of caregivers. This fear often refers to all kinds of sexuality, sexually transmitted diseases, risky contacts, and even curiousness. Many children with ID have a dual diagnose of motor impairment, and may not perceive body signals appropriately (e.g., the need for toileting). Very often, children with ID share private places, such as bathrooms, with other people touching them for adequate reasons. Here, lists of people being allowed to touch children can be compiled, including the circumstances and the location of a touch (when and where). The concept of secrecy as it is used by perpetrators, might be unknown to them. Privacy often has to be taught explicitly as the control over the own body and the right to decide about boundaries, such as closed doors. Routine and rule-based behaviors are more familiar to people with ID than concepts as “privacy” for coping with ambiguous situations. Ambiguous situations may occur when rewards are offered by perpetrators and individual decisions are needed (21). Trainings of preventive skills in individuals with ID, however, should go beyond sexual knowledge and the practicing of appropriate social behaviors (22). For better protection, potential victims need to recognize potentially abusive situations independently, i.e., without the help of a caregiver. Protective behavior can be divided into appropriate responding to abusive-seductive situations and appropriate reporting. In many cases, individuals with ID make decisions in which they must weigh potential harm against real or illusory benefits. Khemka and Hickson (22) concluded for adults with ID that preventive trainings should foster independent, generalizable self-regulatory skills, such as decision-making, assertiveness and self-empowerment. This should be even more applicable to children and adolescents who start to encounter these developmental tasks. Self-assertiveness for children and adolescents with ID means to recognize and trust in one's emotions, to express displeasure, and to stand their ground.

Considering the fact that almost no information on trainings, measurements and ethics was available in advance, many steps had to be developed by ourselves. These steps are described in greater detail below.

### Development of the training

To tailor the intervention to the needs of the target group [as demanded, for example, by (23)], the training was developed in a 4-step process, (1) reviewing literature on programs and evaluation studies on children with ID, (2) conducting focus groups with German experts versed in CSA prevention for children with ID, (3) conducting a pilot study on the feasibility of the intervention and the measurements, and (4) re-working the materials according to the findings [see (15)]. At the end of this process, the training program was developed according to the rationales listed below. Each rationale was turned into exercises which were arranged into 10 sessions, each filled with content, exercises, and methods that are in parts established, in parts innovative. The training's rationale is based mainly on behavioral therapeutic exercises and contains psychoeducational elements. The material will be available soon in the German language and will be provided by the authors upon request.

#### Mix cognitive and behavioral tasks in an appealing way

Limited sexual knowledge is a major barrier in preventing sexual abuse (24–29). However, teaching knowledge on sexuality and prevention alone is insufficient for sexual abuse prevention (24). Combining it with a Behavioral Skill Training (BST), however, is a well-established training method. BST uses role-plays, instructions, modeling, repetition, reinforcement, and corrective feedback. Six evaluation studies speak for the effectiveness of the training method in the context of sexual abuse prevention and with subjects with ID (17, 20, 30–33). The program presented in this study uses a mixture of cognitive and behavioral tasks to fill gaps in knowledge (identifying situations of abuse, *knowing* what to do) and to practice the preventive strategies (*doing* what may be done to prevent or disclose abuse).

#### Use role-plays

Our review on the German and international literature (25) found only four programs available for children with ID. Knowledge from these studies was enriched by expert focus groups and knowledge from studies that has not undergone peer review to establish a manual. Training methods in the peer-reviewed studies vary greatly, but despite their broad range, 3 out of 4 approaches (32, 34, 35). Kim (20) used role-plays as their main method of teaching. All more recent publications (20, 33, 36) also make use of role plays, which have by now been identified as “essential” (20). However, role-plays require all parties to understand and remember who is playing which role, involving complex cognitive skills to differentiate between the immediate situation and the pretend situation. Lee et al. (34) thus urge the combination of role plays with suitable communication aids, which have been implemented in this study in the form of video films and visual prompts complementing verbal instructions and room for discussions. The methods video film, games, group discussions and role-plays were effective and appropriate for children (1) and children with ID (37). Role plays were not only effective in conveying the subject matter but were also shown to remarkably improve the attention span in individuals with ID (17).

#### Cover different situations

As CSA happens in very different contexts and situations, the training aims at covering those by including and randomizing various offenders, contexts, and abusive behaviors (see Table 1).

**Table 1:** Video vignettes used for the training and the measurements, respectively.

	**Location**	**Offender (relationship to the child)**	**Offender's sex (male, female)**	**Offender's age (young, medium, old)**	**Sexual request**
1	Home	Brother	M	Y	Intrusion into privacy (in the bathroom)
2	School	Classmate	M	Y	Kissing
3	Bus	Bus driver, federal voluntary service member	M	Y	Obscene comment (breast size)
4	Girl's room	Cousin/Friend	M	Y	Watch pornographic material, undress (both)
5	Home	Cousin/Friend	M	Y	Take naked pictures of the girl
6	Changing room (school, sport)	Friend/Trainer/Youth leader	M	Y	Undress (have bra opened by the offender)
7	Home	Uncle	M	M	Kissing
8	Home or children's home	Mother's boyfriend or caretaker	M	M	Undress, touch girl's body (apply a lotion after her taking a shower)
9	Home	Neighbor, babysitting	M	M	Fully undress, paint the girl naked
10	Home	Babysitter (parents' friend, possibly neighbor)	M	M	(Fully) undress, examine the body (both actively and passively, acting like doctors)
11	Home	Mother's friend	M	M	Stroke penis (above pants)
12	Bus	Bus driver	M	O	Kissing
13	Home	Grandfather	M	O	Lying in bed and kissing (potentially, wearing underwear only)
14	Playground	Stranger	M	O	Abduction, seizing the girl's arm
15	Home or children's home	Undefined (possibly neighbor, teacher, caretaker, family member)	F	M	Undress, lie down on back, be anointed with oil and massaged
16	Home	Babysitter (neighbor, friend of the parents)	F	M	Be caressed between the legs above the pants, be asked to take off pajama pants
17	School	Classmate	M	Y	Look at penis
18	Home or children's home	Roommate	M	Y	Undress, act out and videotape sexual scenes (produce pornographic content)
19	Home or children's home	Caretaker	M	M	Look at penis
20	Office	Therapist	M	M	Undress (show underwear)
21	Office	Physician	M	M	Undress (take off pants and slip), be touched at the vagina
22	Summer Camp	Caretaker	M	O	View pornographic magazine, watch masturbation
23	Church	Pastor	M	O	Undress
24	School	Teacher	F	M	Caress each other's vagina

#### Use visual aids

As mentioned above, if children with ID are unaware of *why* they are carrying out the behavior requested of them in role plays, little will be learned (34). Children with ID engaging in role-plays on sexual abuse prevention require aids. To this end, video vignettes, sometimes overlaid with verbal narratives, proved useful for adults (38, 39) and children with ID (37). There is experimental evidence that adults with ID can benefit from visual aids in complex decisions (40). Our training combines video clips and modeling the target behavior in the role-plays, guiding participants on how to utilize the newly learned skills. Sequences of behavioral exercises and reflection alternate so that participants can observe the concept at work on themselves or others.

#### Repetition and nature of instructions

Alnahdi (41) summarizes for the attainment of reading skills that “students with intellectual disabilities need to be exposed to extremely intense practice and instruction […] which should be provided explicitly, systematically, and consistently” (41). All sessions use a repetitive framework in an incremental [bottom-up, see (41)] fashion to enhance memory performance. Many concepts were first presented in a rather cognitive yet still play-like manner and are then exercised in a behavioral way, e.g., using role-plays.

#### Group training

Group trainings were applied by the majority of evaluation studies (22, 31, 32, 38, 42, 43). Since the literature review lists evidence for the efficacy of teaching in small groups, in which students are acting as reciprocal models (44), this method was chosen to strengthen the participants' learning effect. One-on-one trainings have been used in the field on very small samples (17, 20, 30) and presented some disadvantages, as they put excessive burdens on staff and effects of social facilitation were missed.

#### Motivation

Miltenberger et al. (17) report a decrease in concentration after 20 min for adults with ID, which was lesser pronounced during role-plays, as compared to seatwork. Concentration has also been shown to depend on the motivation [(45); compare (20)]. The participants of this study were able to actively engage in the training and co-create content (e.g., to name situations from their own experience to be performed in role plays) and were provided with positive identification figures and role models, all of which is known to enhance the motivation of students with ID (44). Within training sessions, more complex tasks were followed by playful group activities wherein girls could move around, relax, or be creative. Social interactions are also a strong factor for their motivation, underlining the advantages of group programs.

#### Empowerment

Another aspect for program development was the perspective of empowerment (46). The participants are taught about their rights and bodily autonomy. They learn to trust their own emotions even when someone tries to persuade them of the contrary. They learn to defend their boundaries in situations of invasion of personal space and sexual abuse. They are also taught that they have the *right* to defend themselves—not the obligation. It is made clear that it is in no case their fault when they become a victim of sexual abuse and/or when they do not execute the steps that they are taught, as it is the grown-ups' responsibility to ensure the children's safety.

#### Modeling

A hand puppet called Emma “assists” the trainer in teaching the concepts [see (47)] serving as an “advanced coping model.” While so-called mastery models exclusively demonstrate the successful mastery of a requirement, coping models also include problems that may occur along with the successful handling of a challenge. While studies on adults without ID showed coping modeling being superior to mastery modeling [e.g., (48)], the efficacy of coping modeling in children has been criticized (49). The Emma puppet was conceptualized as a mixture of a coping model and a mastery model, allowing the girls to imitate the model and still develop their own strategies and insights.

### Development of video sequences

The kind of CSA greatly depends on the situation, i.e., the institutional framing or gender and age of the perpetrator. According to Brown and Turk (50), it includes “but [is] not limited to, verbal harassment, unsolicited touching, penetration, and forced viewing of inappropriate sexual material” [(16), p. 168]. The inclusion of non-contact sexual abuse (50) into the training was also a demand expressed by experts in the focus groups. In this study, we tried to control for situational effects by covering the spectrum *via* different video vignettes used during the training and during measurements randomizing the latter to participants and points of measurement. Twenty-four vignettes were produced in total (see Table 1), out of which 16 were used during the training sessions and the remaining 8 were presented only during measurements (2 at each point of measurement). The latter were stopped before the lure or request was presented, whereas the training vignettes were shown in full, which included the girl's exemplary response. For the vignettes, we tried to represent distributions of situational factors as they were described in the literature (30) and the focus groups. Most of the perpetrators are male (18, 19, 51) with their age grouped into three blocks (around 18, 25–35, 55–60 years). Vignettes lasted about 90 seconds and described situations at the family home, institutional living quarters, public spaces during free-time activities (sport, summer camp, church), school, and disability-related situations (therapist, doctor, transportation). Video clips not only contain seductive situations preceding abuse as described by Sobsey and Doe [(12), see above], but also include other forms of sexual abuse. Physical violence was left out for the most part because experts argued that these situations were easily detected as abusive. Vignettes for measurement (Table 1, 17–24) were randomized across persons in a way that two different vignettes were shown per person and measurement.

### Dependent measures: Preventive knowledge and behavior

**Figure 1:**
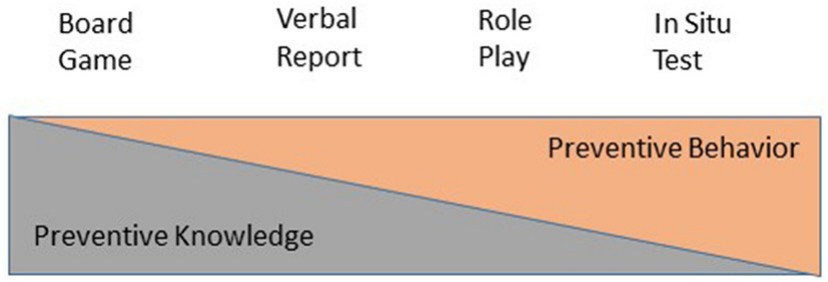
Ordered sequence of dependent measures of prevention skills, composed of two theoretical constructs.

In summarizing advice given by several authors, we grouped dependent measures heuristically into preventive knowledge and preventive behavior (see Figure 1). We assumed them as being ordered on a dimension ranging from “pure” knowledge to “pure” behavior bearing in mind that these distinctions need to be researched further. As the “purest” form of preventive knowledge we regarded *cognitive representations* of names (private parts, emotions), differences between appropriate and inappropriate touches and subjective rationales (e.g., if a touch is “disliked”) and protective behavior (i.e., *knowledge* of protective behavior). Items were extracted from the Personal Safety Questionnaire (52), from a program on adults with ID (53), from a guidance published in Germany (54) and discussed within focus groups with experts from the field. Nineteen items were presented in the standardized form of a *board game* with no feedback given on the accuracy of responses. Scores could range between 0 and 25 (materials can be obtained by the corresponding author). As preventive knowledge, but somewhat closer to reality, answers to “what if” situations were recorded. Short video clips were presented to participants who were asked for their behavior if they were in the same situation. Video clips were produced for the purpose of the study, similar in actors and surroundings to the clips used during the training, but different in situational settings.

Two *Verbal reports* per measurement given by the girls were videotaped and rated by three independent blinded raters on a scale developed by Lumley et al. (31) for adults. Three independent blinded raters scored preventive behavior along a rank order that started at the lowest score of 0 for any occasion in which the girl complied to the seduction or showed no other kind of behavior. A score of 1 was given for “does not agree to engage in the requested behavior (appraisal)”; a score of 2 for “says ‘no' or otherwise verbally refuses (refusal)”; score of 3 for escape behavior, such as “leaves the situation or tells the offender to leave”; and a score of 4 for “reports the incident to a staff person.” After discussion with experts from the fields, the team agreed that preventive skills in this chain are neither fully hierarchically ordered nor logically disjoint. Instead of an “either-or,” girls might rather use different strategies in one act. Therefore, we decided to use an additive score for preventive behavior ranging between 0 and 10. The same score was used to assess preventive skills in *Role Plays* one step closer to reality. Instead of imagining situations girls had to play their responses to played seductions. Again, two role plays per measurement were videotaped and rated by three blinded raters each with the scores averaged. Situations were played by the girls who imitated models from training videos with the trainer playing the offender. To signify her change of role the trainer changed her outfit by wearing a hat or cap. At least, so-called *In situ* tests [e.g., (55)] came as close to reality as we could get. Hired actors played the offenders with girls not knowing being deceived. For locations the team had to make the best of what was available, such as quiet rooms in schools, medical practices, waiting rooms and hallways. Again, preventive behavior was rated on a scale ranging from 0 to 10 points maximum.

### Measurement of covariates

Extracting hypotheses from the literature we assumed different confounding variables to have notable effects on the change of preventive skills *via* training. First, intellectual disability might vary greatly among girls fostered in special schools. ID was assessed by the general IQ which had to be measured by a renowned instrument. In case no IQ was found in the school or personal records the Wechsler nonverbal scale of ability (56) was conducted. Aside from ID, grammar comprehension was measured by the raw sum score of the Trog-D (57), a German test measuring important grammar concepts, such as the subject-object discrimination. Comorbid mental health problems were assessed using the Child Behavior Checklist [CBCL, (58)]. Sexual maturity was assessed with the parent's judgements based on the widely used ratings for secondary sexual characteristics (59). Rater's judgements were combined as averages, (a) across two different situations in Verbal Reports und Role Plays and (b) across all three raters. Variations around rater's means were calculated as approximations for their agreement.

### Ethical considerations and safety

In 2010, three German ministries (Justice; Family, Seniors, Women and Youth; Education and Research) established a “Round Table” (Runder Tisch) on CSA after reports of CSA within the Catholic Church and other institutions had shaken the republic. Headed by the ministries, a work group was established and an independent commission for the reappraisal of CSA events in institutions and families started to work. Since then, an independent federal commissioner (Beauftragter für Fragen des sexuellen Kindesmissbrauchs) gathers and manages complaints and reports. In their Final Report (60), the team of the Round Table demanded to establish prevention measures on all levels, including the delivery of preventive knowledge to children, parents and others (p. 38). The Ministry of Education and Research therefore established a research network by a public competition in health research (61). The study presented here was developed as part of this network, which met regularly for presentations of ideas, lessons learned, results, and discussions on ethical issues. The major ethical consideration of our study regarded the justification of the *in situ* tests. Focus groups described the dilemma of needing interventions as close as possible to everyday life on one hand but minimizing possible harmful effects to learners on the other side. We were in a special need to minimize type II errors: A *wrong* assumption that a prevention training for CSA would be successful has a potential to *harm* subjects if caregivers falsely believe their fosterlings to be protected. For that reason, we established *in situ* tests. To ensure the participants' wellbeing, we simultaneously widened the definition of a potentially adverse event for both the measurements as well as the training. All cases in which participants and/or their parents wanted to leave the study for reasons of incommodity or negative feelings were coded as adverse, independently of the severity of the event.

#### Standard operations procedures and ethics approval

Standard operating procedures (SOPs) for potentially adverse events during the intervention and measurement were established and events were recorded. For the *in situ* tests, the SOPs included scripted guidelines for the actors and for the preparation of locations, such as comparable means of escape for the participants and the setup of the video camera. All training sessions and assessments were videotaped to allow for later and/or independent inspection. During all assessments, a trusted adult waited outside the test room to offer immediate emotional support.

In their schooling prior to the study's implementation, trainers were sensitized to adverse events and instructed what to do if they observe unusual behavior or if the child discloses abuse during the study. Such events and the steps that were taken were documented carefully.

Approval of the study was given by the Ethics Committee of the University of Rostock and the federal state government of Bavaria.

#### Consent and counseling

After the school administrations had given consent to incorporate the study's interventions into their school routine, parents were informed in detail about the study and gave written consent on behalf of their offspring for their daughters' participation in the intervention and in the evaluation. In presence of their parents, girls were informed about the rough content and outline of the intervention. They were explicitly and repeatedly informed about their right to pause, skip or discontinue the sessions at any time. Participants who expressed distress during or after the sessions or measurements were approached one-on-one by the trainer who sensitively tried to assess the situation. If the trainer felt the necessity, girls were then asked whether they wanted to share their feelings with their parents and/or with counselors. Both centers cooperated with local contact points for girls and women who have experienced sexual violence to receive further counseling if necessary.

#### *In situ* tests

*In situ* tests were carried out following a standardized procedure: (1) Girls were situated in a room, separately from a care person who was close by (mostly parents who waited for their daughters in the hallways). The girls were informed that they were being videotaped in the beginning of the assessment; the video recorder was visible to the girls at all times. (2) The trainer initialized an interaction (such as drawing together) and left the room using an excuse. (3) After several minutes, the actor who played the offender entered the room using a standardized excuse, such as waiting for his own daughter. (4) After a standardized warm up (drawing from various options, such as asking about the drawings), the offender asked the girl to do him a favor, such as kissing or pulling off the clothes; these requests were scripted and always used the same wording. (5) In case the girl started to comply with the favor, the actor immediately interrupted the situation using an excuse, such as having a call from somebody, and left the room; in case the girl refused, the actor politely accepted the refusal left the room in a timely manner; in case the girl did not react, the actor waited for 30 s (to allow time for the girl to refuse) and then politely excused himself and left the room. (6) The trainer re-entered the room after a while and continued the previous interaction for 5 min to allow time for the girl to report the situation. (7) The trainer ended the situation and returned the girl to her parents. 8) In case the girl had not reported the incident to the trainer, parents were asked if the girls had reported the incident to them.

## Hypotheses

The primary hypothesis of this study was that gains in preventive knowledge and behavior would be bigger in the intervention group compared to the control group. Gains in preventive knowledge and behavior should last for at least 3 months. Secondarily, we were interested in the influence of different covariates, both content-related (intelligence, grammar comprehension, sexual maturation, behavior problems) and methodical (rater's agreement, center-effects).

## Methods and materials

### Recruitment

**Figure 2:**
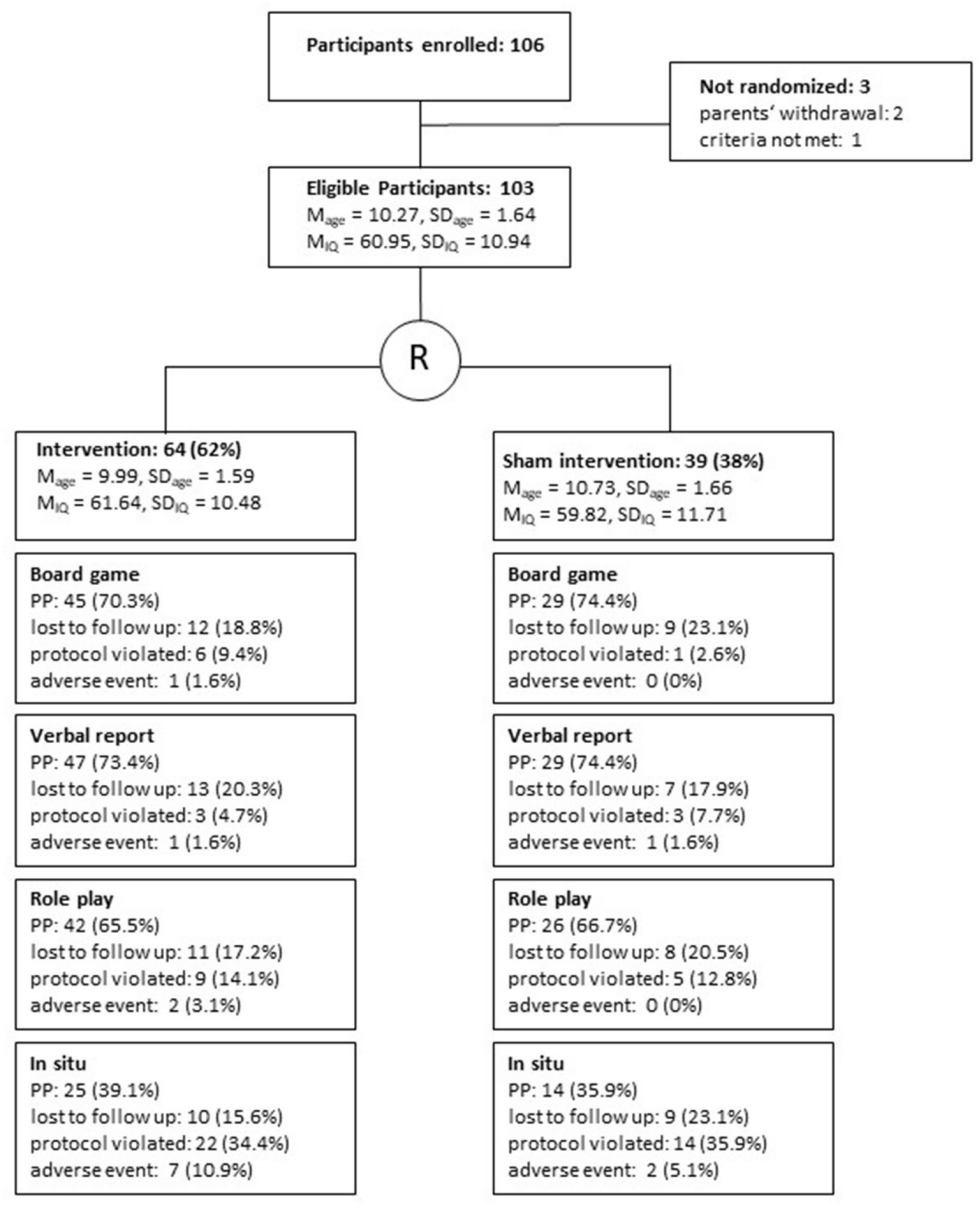
Study flow for the evaluation of Emma-Untouchable, with drop-outs for different outcomes, reported at any time during the investigation, M, Mean; SD, Standard deviation; IQ, Intelligence Quotient; PP, Per protocol sample t_1_ – t_3_.

Girls were recruited at 14 schools and care centers in two German states, Mecklenburg-Pomerania and Bavaria. After obtaining the schools' consent, parents were informed in detail and signed informed consent. Inclusion criteria were age (8–12 years) and intellectual disability (50 ≤ IQ ≤ 70, or a comparable severity of cognitive disabilities). As stages of development vary greatly in the target population, some older girls were included (for justifications, refer to the register of the study, DRKS00014673). Girls were excluded if they suffered from a pervasive developmental or a severe and acute psychiatric disorder. Of the 106 girls enrolled to the study, two withdrew consent before the first measurement and one participant was excluded because of a severe psychiatric condition, leaving 103 participants to be cluster-randomized (based on their school affiliation) to either one of the experimental conditions (see Figure 2) by an independent institute (IBIMA, Rostock). About 62% (*n* = 64) of the eligible sample were assigned to the intervention group, leaving 38 % (*n* = 39) for the control group.

In most cases, parents took their daughters to the location of measurement, thus receiving 50 € travel reimbursements at the beginning of the study and 100 € after completion of the last measurement.

### Control group's training program

The control group received a training program aiming at preventing accidents and enhancing traffic safety. Thus, both prevention trainings differed in content but were equal in length, familiarity with the trainer, and methods. For example, the control program incorporated Emma the doll and introduced and implemented role play just as the intervention program did. This way, we made sure all girls had practiced this type of exercise before the second point of measurement.

### Sample

The sample consisted of 103 girls aged 10; 4 years (SD = 1.64) on average at the time of the first measurement. Groups differ significantly in age (see Figure 2, average difference = 9 months, 95% confidence interval, ranging from −16.7 months to 10.2 months, *p* < 0.05). On average, girls had an IQ of 61 (SD = 10.94) measured *via* different established tests. Groups do not differ in terms of intelligence (average IQ difference = 1.8 points, 95% confidence interval: −2·58 to 6.24 points, *p* > 0.05).

### Safety measures

Standard operating procedures for potentially adverse events during the intervention and measurement were established and events were recorded. All cases in which participants and/or their parents wanted to leave the study for reasons of incommodity or negative feelings were coded as adverse, independently of the severity of the event.

## Analysis

The change in preventive knowledge and behavior was modeled as the dependent variable in four mixed models adjusted for the respective baseline values. Intelligence (IQ), age, instruction comprehension (raw score Trog-D), psychiatric burden (general score CBCL), raters' agreement (standard deviation of three ratings) were modeled as covariates, and center, time, experimental condition, and the interaction between time and condition as fixed effects. The different measuring points of a participant were incorporated as a repeated effect with a first order autoregressive covariance matrix. As the study had to be ended earlier due to dwindling resources the intervention group turned up with a somewhat larger sample (see Registry DRKS00014673). This way the likelihood increased that possible confounders, such as intelligence, age, instruction comprehension, center and psychiatric burden were not fully controlled for. We decided to model them as covariates and to cross-check for models without covariates. In this paper we present only models with covariates and describe deviations in case they occurred within models without covariates. Doing so we will get information about effects of possible confounders described in the literature and exclude their effects on group differences. These analyses were done with the intention-to-treat population and repeated for the per-protocol population. The results are presented using model-based means and their 95% confidence intervals in tables. To investigate the assumptions of a mixed model, we used histograms of the residuals. Drop-out rates for three reasons (lost to follow up, protocol violated, adverse event) were compared for both groups using Fisher's exact test. The significance level will be set at 5% (two-sided). All statistical analyses were conducted with SPSS, version 24 (IBM Corp, Armonk, NY, USA).

## Results

Fisher's exact tests calculated separately for all dependent measures revealed no differences in drop-out rates (see Figure 1) for the Board Game (χ^2^ = 2.34, df = 2, *p* > 0.05), Verbal Reports (χ^2^ = 1.14, df = 2, *p* > 0.05), Role Plays (χ^2^ = 1·07, df = 2, *p* > 0.05), and *In Situ* testing (χ^2^ = 1.68, df = 2, *p* > 0.05). The amount of violated protocols and adverse events increased slightly from preventive knowledge to preventive behavior, peaking for *In Situ* tests (see Figure 1). The report of results follows the sequence from knowledge to practice, starting with the Board Game and ending with *In Situ* tests. Results are presented as difference-scores referred to the baseline (t_1_). Results are reported for the mixed models, which equals an intention-to-treat approach. If results from per-protocol analyses differ from those, they are reported accordingly.

Board Game: Between-group differences in favor of the intervention were observed for the time after training (t_2_, see Table 1) and 3 months later (t_3_). The analysis revealed no significant interaction of time and intervention (*F* = 1.46, df = 1, *p* > 0.05). Of the covariates included in the model, comprehension of grammar (*F* = 12.74, df = 1, *p* < 0.01), and center (*F* = 4.59, df = 1, *p* < 0.05) had significant effects.

Verbal Reports: Between-group differences are of statistical significance in favor of the intervention for the time after training and barely miss the threshold of 5% for the time 3 months later. If covariates are taken out of the model this difference is statistically significant. Throughout the analysis this is the only relevant difference between models with and without covariates. The interaction of time and intervention is significant (*F* = 5.55, df = 1, *p* < 0.05) and grammar comprehension is the only covariate of statistical significance (*F* = 6.50, df = 1, *p* < 0.05). In the analysis per protocol, the difference between groups after 3 months is statistically significant.

Role Plays: The change of preventive behavior as displayed in Role Plays differed significantly between both groups favoring the intervention but did not last until 3 months later. The interaction of time and intervention was not significant (*F* = 3.52, df = 1, *p* > 0.05). Among the covariates, the center (*F* = 7.44, df = 1, *p* < 0.01) had a strong effect, as well as the raters' agreement (*F* = 11.71, df = 1, *p* < 0.01).

*In Situ*: The between-group difference for the time after training is statistically significant in favor of the experimental condition, an effect lost within the per-protocol analyses. The between-group difference for the third measurement is not significant, which is due to both a loss within the intervention group but also a small gain in the control group starting during the traffic-safety training and lasting for the time after intervention. The interaction of time and intervention reached statistical significance (*F* = 4.36, df = 1, *p* < 0.05). None of the covariates proved to be significant. While the intervention group in the intention-to-treat analysis maintains a significant gain 3 months after training when compared to the baseline, this effect is lost if cases with protocol violations are excluded (Table 2).

**Table 2:** Changes in prevention knowledge (Board Game, Verbal Report) and prevention behavior (Role Play, *in situ*) for intention to treat analysis, baseline adjusted measures, Baseline—before intervention, t1—after intervention, t2—3 months after intervention, Range Board game 0–25, Ranges other measures 0–10.

	**Intervention group**							**Control group**							**Between group differences**				**Interaction between**
	**Change from baseline**							**Change from baseline**							**Intervention group—control group**				**group and time**
**Outcome** **variables**	***N***	**Mean**	**SD**	**Adjusted** **mean**	**95% CI**		***p***-value	***N***	**Mean**	**SD**	**Adjusted** **mean**	**95% CI**		***p*** **-value**	**Adjusted** **mean**	**95% CI**		***p*-value**	***p*-value**
Board game																			0.2300
Baseline	63	6.66	3.29					35	7.25	3.34									
t_1_	58	10.82	4.22	4.04	3.30	4.78	<0.0001	39	7.47	3.35	0.27	−0.72	1.25	0.5909	3.77	2.51	5.04	<0.0001	
t_2_	55	10.69	4.12	3.68	2.93	4.44	<0.0001	39	7.71	3.53	0.53	−0.45	1.52	0.2850	3.15	1.88	4.42	<0.0001	
Verbal report																			0.0210
Baseline	64	1.96	1.94					36	2.27	1.84									
t_1_	61	4.01	2.63	2.00	1.50	2.50	<0.0001	39	2.35	2.18	0.06	−0.62	0.74	0.8690	1.94	1.08	2.80	<0.0001	
t_2_	54	3.34	2.39	1.19	0.66	1.72	<0.0001	39	2.69	2.53	0.32	−0.36	1.00	0.3528	0.87	−0.01	1.75	0.0520	
Role play																			0.0640
Baseline	63	1.59	1.39					35	2.05	1.48									
t_1_	61	2.57	1.81	1.04	0.67	1.41	<0.0001	36	2.02	1.41	−0.04	−0.56	0.48	0.8721	1.08	0.43	1.74	0.0010	
t_2_	52	2.07	1.55	0.63	0.23	1.03	0.0020	36	2.16	1.69	0.22	−0.30	0.74	0.4050	0.41	−0.23	1.03	0.2260	
*In situ*																			0.0410
Baseline	52	2.03	2.06					35	2.57	1.99									
t_1_	45	3.35	2.45	1.56	0.87	2.26	<0.0001	34	2.80	2.15	0.41	−0.39	1.21	0.3099	1.15	0.05	2.25	0.0400	
t_2_	42	2.80	2.29	0.89	0.17	1.60	0.0152	34	3.49	2.22	0.96	0.17	1.74	0.1720	−0.07	−1.16	1.02	0.9000	

*SD, Standard Deviation; CI, Confidence interval; p, Significance*.

## Discussion

This study tried to provide insights on effects of a sexual abuse prevention program on girls with ID on the highest quality of evidence possible to us. For the first time to our knowledge a sufficient sample of girls with ID was trained with a state-of-the-art program and examined within a randomized controlled trial with an active control condition. Dependent variables were ordered on a theoretical continuum (see Figure 1) according to their implementation of knowledge and behavior.

For the “pure” knowledge measured by the number of correct answers in a *board game*, small but lasting gains were observed. Grammar comprehension was a significant covariate, signaling its importance for understanding and answering the items of the board game. Rather complicated questions such as “Who may decide whether someone is allowed to give you a kiss or to touch you?” or “What can you do if an adult threatens you or wants to kiss you even though you don't like it?” needed to be understood fully. The complexity of potentially abusive situations, however, requires basic grammar skills, such as to distinct between object and subject or between active and passive verbal constructions. Significant effects of the study center may have occurred since instructors or situations may have differed slightly during the measurements. While board games in Munich were played mostly at schools with sometimes noisy backgrounds, they were administered in Rostock in situations closer to laboratory conditions.

*Verbal reports* answering the question “How you would react in that situation” after a video-exposition were measured on a different scale. This scale ranged from 0 up to 10 points with a maximum of protective behavior. An observed change of nearly two points, if started from zero, means a change in behavior from “does not engage” to “actively refuses,” which seems to be a small step numerically, practically however makes a lot of a difference. Here, girls have learned to become actively involved and that they might control an abusive situation. Both groups started from active refusal (score around 2) with the intervention group learning to protect themselves somewhat more (ending a situation themselves, score around 4). This effect decreases after 3 months (score 3.34) but remains significant when compared to baseline. The control group did not show any of these changes but does not differ any longer from the intervention group when a trimester has passed. Again, grammar comprehension makes a significant covariate which is obvious when self-reports about imagined situations are examined.

*Role plays*, in our view come one step closer to reality, when compared with verbal reports. Within the intervention group preventive behavior increases by one point (from 1.6 to 2.6) which equates a move from rather passive to more active behavior. The loss after training however is bigger compared to verbal reports. Again, we observed a significant change from baseline but no difference to the control group. Also again, no changes within the control group speak for a special effect of the training. For verbal reports strongly covarying variables were observed. The effect of the center is interpreted as a trainer effect. In one center, the trainer felt uneasy playing the offender, even if she transformed herself noticeably, e.g., by wearing a hat. We interpret these finding as a call to enhance role plays. A good, but costly opportunity is to have an actor available playing the intruder. Another option would be to use individual video recordings of earlier successful refusals. Role plays seem to be harder to rate, as the significant covariate of the raters' agreement indicates. Aside from trainer effects this might be due to the concept of role-play itself that was not well-understood by a part of our participants according to our observations. As indicated by our results this kind of variance was not explained by the intelligence, grammar comprehension or psychiatric symptoms. It rather may be that girls with ID may have different experiences with roles, role play or even watching role models on a screen. A more individualized program of the future should carefully watch and deal with the scruples participants may have to act in “as if” situations.

*In situ* tests are coming as close to everyday life as one can get, with their validity arguably outweighing the shortcomings described before (31). As no comprehension for the situation was induced it seems plausible that no effects of covarying variables were found here. An interesting effect was observed for preventive behavior in naturalistic situations. For the control group we found a steady increase (2.57–2.80–3.49) while the intervention group lost half of its initial gain (2.03–3.35–2.80) after 3 months. As calculated, the intervention itself had no effect and groups did not differ at all after adjustment for baseline effects. For the interpretation of this result, we had to change our view. It may be that the group training did not make a big effect after all but that girls from the control group may have learned from the naturalistic exposition during measurements. *In situ* tests were performed within this group three times, too, turning them perhaps into a kind of training. Situations as sitting alone somewhere in a waiting room, a school, or a park bench may be stressful for girls not used to it. The appearance of strangers trying to lure girls to do something unwanted was assumed to be a major stressor at the beginning of the study. Sophisticated SOPs how to deal with different outcomes of these situations were set up in order to buffer all kinds of stress likely to occur. On the other side, this reality-stress may have heightened the girls' arousal facilitating the connection of learned behavior and emotions. The heuristic model of preventive skills perhaps needs to be supplemented by the dimension of “emotional involvement” or “awareness.” “Dealing with emotions of uneasiness” was a module of the program, which perhaps needs to be trained more intensively. Future modifications of preventive trainings with girls with ID could include naturalistic setting and emotions directly involved in the learning process. However, how subjects with ID process emotions is a widely unresearched area (62), which makes it difficult to give recommendations here.

More hints for modifications of the training, the measurement of effects or the modeling were derived from the inclusion of covariates. Both, methodological and content-related covariates were shown to have effects on the results. For the training it means, that comprehension of training contents should be closely monitored. Perhaps, group sessions should be supplemented by individual sessions where issues as comprehension or emotionality are reworked in greater detail. As for measurements, the in- or exclusion of covariates did not change the results of this study much. However, possible confounders, such as raters‘ agreement, center-effects, or comprehension of grammar should be controlled for thoroughly in studies to come. It may well be that constructs as grammar comprehension play a mediating or moderating role in linking preventive efforts and effects. However, analyses of this kind were beyond the scope of this paper.

In summing up our results we recur to the ordinal sequence of our dependent measures and postulate: The closer the measurement of preventive skills comes to reality the smaller the effect of a person-centered training will be. Preventive knowledge hardly translates to preventive behavior. In our view, this study convincingly emphasizes the need for multi-method and ecologically valid measurements in studies with participants with ID.

Moreover, effects observed stayed small after all. In relation to the best possible preventive behavior (a maximum score of 10 points), including not only refusal, but also active ending and reporting, the changes in the intervention group were only marginally sufficient. The level of “active ending” a situation (score > 4) was not reached for any of the outcomes. Our team concluded that girls with ID were not sufficiently protected by solely completing the program “Emma—Untouchable.” To do that, prevention needs to go beyond the individual level up to the situational or institutional level.

Several authors have argued that prevention needs to move from programs to systems (10) not only targeting individuals but also circumstances and the interaction of both. People with ID are a highly vulnerable group ideally protected by a closely knit network which they learn to navigate on one side and wherein they learn to live a self-determined sexuality on the other side. An effective way to do this is to sensitize and empower caretakers as well (63).

In trying to accomplish a level of evidence known from clinical studies also for prevention research, our work provided some lessons about the field of children and adolescents with ID. First, sufficient samples for prevention research are perhaps harder to recruit from all sorts of institutions when compared to clinical wards. Sufficient sample size could not be gained during the first turn of the study presented here, making a second wave of recruitment necessary. For the second wave both centers extended their search space beyond municipal boarders. Many problems needed to be mastered within this outreach work, such as the transport of parents, children, and actors playing offenders to very different places. Second lesson was, that ecological validity, as it is essential for prevention research, cannot be achieved in the lab (64). Real life-situations, however, are hard to standardize. Protocols for measurements (SOP) need to be established carefully during extended pilot studies. About one third of *in situ* measurements were not rated because of protocol violations indicating all sorts of problems. Nonetheless, adverse events occurred equally frequent in both groups. We interpret this result as (1) no additional effect of the training on the likelihood of unwished events, (2) an effect of providing a safeguarded sham intervention, in which participants encountered the same trainers for an equally long period of a closed relationship. As closer the measures of interest came to real life, the more protocol violations were recorded throughout the examination, for several reasons. For Role Plays, some trainers were uneasy to play the offender ending up with non-adherent behavior, such as wrongly used instructions, and the like. For *in situ* tests, it was often hard to find actors and to transport them to locations, some actors did not follow the SOP correctly, some investigations were disturbed by other intruders, such as teachers. Again, these violations occurred equally frequent in both groups.

## Data availability statement

The raw data supporting the conclusions of this article will be made available by the authors, without undue reservation.

## Ethics statement

The studies involving human participants were reviewed and approved by Ethics Committee of the University Medical Center, Rostock. Written informed consent to participate in this study was provided by the participants' legal guardian/next of kin.

## Author contributions

OR, FH, and WC designed the study. WC conducted the study. AD and OR conducted data analyses. OR, FH, AD, and WC drafted the manuscript. All authors contributed to the article and approved the submitted version.

## Funding

The German Ministry funded the whole project (Grant number 01KR1206) and had no role in conducting research or publication. The funding by the ministry includes publication fees.

## Conflict of interest

The authors declare that the research was conducted in the absence of any commercial or financial relationships that could be construed as a potential conflict of interest.

## Publisher's note

All claims expressed in this article are solely those of the authors and do not necessarily represent those of their affiliated organizations, or those of the publisher, the editors and the reviewers. Any product that may be evaluated in this article, or claim that may be made by its manufacturer, is not guaranteed or endorsed by the publisher.
